# Unveiling the underestimated prevalence of HIV, HBV and TB triple infection in Asia, South America, and Africa: a systematic review and meta-analysis

**DOI:** 10.1186/s12879-025-12390-4

**Published:** 2025-12-20

**Authors:** Yinkang Mo, Zihao Fan, Yaling Cao, Ling Xu, Xiangying Zhang, Feng Ren

**Affiliations:** Beijing Institute of Hepatology/Beijing Youan Hospital, Capital Medical University, No. 8, Xitou Tiao Road, Youwai Street, Fengtai District, Beijing, 100069 China

**Keywords:** HIV, HBV, TB, Triple infection, Infectious diseases, Epidemiology, Meta-analysis

## Abstract

**Background:**

Human immunodeficiency virus (HIV) is a genus of retroviruses that targets immune cells and may eventually cause immune-deficiency illness. Triple infection of HIV/hepatitis B virus (HBV) /tuberculosis (TB) leads to a worse prognosis than mono-infection. We aimed to unveil the pooled estimation of HIV/HBV/TB co-infection prevalence in Asia, South America, and Africa.

**Methods:**

A systematic literature search in PubMed, Embase, and the Cochrane Library was performed for studies of the prevalence of HIV/HBV/TB triple infection published from January 1, 1990, to March 31, 2024. The Freeman–Tukey random effects model was used to calculate the pooled prevalence.

**Results:**

We included 7 studies with in total 6401 participants and 259 triple infection cases. The pooled triple infection rate in the enrolled population was 4.4% (259/6401; 95% CI 2.2%-7.3%). The results of the subgroup analysis showed that the prevalence of triple infection was significantly higher in the South America (82/950, 8.6%; 95% CI 6.8%-10.4%), in men (86/1817, 3.4%; 95% CI 0.7%-7.8%) and patients receiving antiretroviral treatment (125/1611, 7.4%; 95% CI 5.8%–9.2%). However, no significant difference in the triple infection rate was observed among individuals initially diagnosed with HIV or TB.

**Conclusions:**

This meta-analysis suggests that the prevalence of HIV/HBV/TB triple infection in the regions studied is underestimated, and we should focus more effort on improving novel strategies for identifying triple infection of HIV/HBV/TB. However, additional studies are required to be incorporated in future analyses to provide a pooled estimate of the global pooled prevalence.

**Clinical trial:**

Not applicable.

**Supplementary Information:**

The online version contains supplementary material available at 10.1186/s12879-025-12390-4.

## Introduction

Human immunodeficiency virus (HIV), persisting as a significant global public health challenge, is further delineated into two principal species: HIV-1 and HIV-2. The progression of HIV infection to acquired immunodeficiency syndrome (AIDS) has hitherto claimed 40.4 million lives, with ongoing transmission observed worldwide. By targeting CD4 surface receptors, HIV attains access to CD4 + T lymphocytes, facilitating the integration of viral RNA into the host cell genome and gradually causing compromised immune functionality [1, 2]. Consequently, individuals with HIV are rendered more susceptible to invasive viral infections, such as TB and viral hepatitis.

Tuberculosis (TB) is caused by the bacterium *Mycobacterium* tuberculosis. It primarily affects the lungs but can also spread to other organs, making it a complex and multifaceted disease. According to the WHO Global Tuberculosis Report 2024, in 2023 an estimated 10.8 million individuals worldwide (95% UI: 10.1–11.7 million) developed TB and approximately 1.25 million deaths were attributed to the disease. Among these, 161,000 deaths occurred in people living with HIV, underscoring the heightened vulnerability of this population [3]. The emergence of drug-resistant TB strains is a rapidly escalating global concern [4]. Recent meta-analyses have elucidated a pooled prevalence of HIV/TB co-infection at 6.0% in China, with a corresponding survival rate of 20.9% for such co-infected individuals. The co-occurrence of TB/HIV substantially elevates mortality risk, nearly doubling that of individuals solely afflicted by HIV [5, 6].

As reported in WHO Global Hepatitis Report 2024, an estimated 254 million people, representing about 3.3% of the world’s population, were living with chronic hepatitis B virus (HBV) infection in 2022 [7]. Given shared transmission routes with HIV, reports of HBV/HIV dual infections abound. Chronic HBV progresses more rapidly to cirrhosis, end-stage liver disease, and liver cancer in the presence of HIV co-infection compared to solitary HBV infection [8, 9]. Globally, the prevalence of HIV-HBsAg co-infection stands at 7.6% (IQR 5.6%-12.1%), with HIV-infected individuals exhibiting a 1.4 times higher likelihood of HBV infection relative to HIV-negative counterparts [10]. Notably, co-infected HBV patients undergoing TB treatment with hepatotoxic drugs such as ethambutol and rifampin experience inferior treatment outcomes [11]. The global prevalence of HBV/TB coinfection stands at 7.1%, with evidence indicating that hepatitis B exacerbates tuberculosis severity [12]. While HBV does not appear to affect HIV or TB responses to antiretroviral therapy (ART), ART in HBV-positive individuals may increase the risk of liver dysfunction, necessitating more frequent retreatment and presenting a therapeutic challenge [13].

Given the substantial public health burden posed by these concurrent infections, comprehending the incidence of triple infection is imperative before delving into underlying mechanisms. Although extant literature has shed light on the estimated prevalence of dual infections between TB/HIV, TB/HBV, and HBV/HIV, a systematic meta-analysis addressing the prevalence of triple infection remains conspicuously absent. To address this lacuna in the scholarly discourse, the present meta-analysis aims to ascertain the prevalence of triple infection through highly reliable references reporting the incidence across diverse geographical regions.

## Materials and methods

This systematic review and meta-analysis was conducted according to the Preferred Reporting Items for Systematic Reviews and Meta-Analysis (PRISMA) guidelines, and it was registered in PROSPERO (CRD42024539864).

### Search strategy

A comprehensive systematic literature search was conducted through PubMed, Embase, and the Cochrane Library databases, for studies reporting TB/HIV/HBV triple infection prevalence and published from January 1, 1990, to March 31, 2024. The full search strategies are displayed in Supplementary Table 1.

### Selection criteria

#### Inclusion criteria

We set 1990 as the starting point for the literature search for the following reasons. The introduction of HBV vaccination after 1990 likely reduced infection rates, and the coexistence of vaccinated and unvaccinated populations may have introduced heterogeneity [14, 15]. Meanwhile, the rapid expansion of the HIV pandemic in the early 1990s led to sharp increases in both global TB incidence and HIV–TB co-infection [16]. In response, the WHO declared TB a global public health emergency in 1993, which prompted strengthened surveillance and the integration of HIV–TB management programs [17].

Studies were selected based on the following inclusion criteria: (1) participants had laboratory-confirmed TB (tested by sputum AFB microscopy and mycobacterial culture), HIV (tested by enzyme-linked immunosorbent assay or western blot method), and HBV (defined as HBsAg positivity) infections; (2) all infections meet internationally standardized diagnostic criteria, with HIV diagnosed by detection of HIV antibodies and/or antigens using a validated two-test (ELISA or rapid tests); HBV infection defined by persistent HBsAg positivity for at least 6 months and/or detectable HBV DNA; and TB confirmed by sputum smear microscopy, culture, or WHO-endorsed rapid molecular tests such as GeneXpert MTB/RIF [18–21]; (3) cross-sectional or cohort studies were collected to estimate the prevalence of triple infection.

#### Exclusion criteria

Studies were excluded if they (1) were case reports, editorial commentaries, review articles or duplicates, (2) had total sample sizes below 50 participants (insufficient for meaningful analysis), or (3) had incomplete or unclear study details.

Two independent reviewers conducted the screening process by performing both title/abstract review and full-text assessment of potentially eligible studies. Any disagreement in study selection process was resolved through discussion and, when necessary, reassessed by a third senior researcher to reach consensus. Both inclusion and exclusion criteria were ensured throughout the screening process.

### Data extraction

Two investigators independently performed blinded data extraction. The collected variables encompassed the study information, type of study, geographical information, age or sex distribution, ART status, total number of enrolled participants and confirmed TB/HIV/HBV co-infection cases.

### Quality assessment

The quality of the selected articles was assessed using the Newcastle–Ottawa Scale (NOS) guidelines (Supplementary Tables 2 and 3), which consist of eight items and yield scores ranging from 0 to 9 stars. Studies scoring more than 5 stars were considered high quality, while those scoring 0–4 stars were considered low quality [22]. Reviewers assessed study quality and resolved any disagreements through consensus.

### Statistical analysis

We used Stata.17 software (StataCorp. 2021. Stata: Release 17. Statistical Software. College Station, TX: StataCorp LLC.) for the systematic review and meta-analysis. Commands used in our manuscript are shown in Supplementary Table 4. To quantify study heterogeneity, we employed the I² index with significance thresholds. p-value < 0.05 was considered statistically significant for heterogeneity testing. When substantial heterogeneity was detected (defined as I² >50%), we implemented the Freeman–Tukey random-effects model to generate pooled prevalence estimates with corresponding 95% confidence intervals. Subgroup analysis was conducted to explore the source of heterogeneity according to different factors, such as different continents, sexes, sample sizes, type of studies and population and ART status. The publication bias was evaluated by Egger linear regression method combined with the observation funnel plot. When studies provided medians and interquartile ranges instead of MDs and SDs, data transformation was performed based on previous statistical research to derive MDs and SDs [23].

## Results

### PRISMA flow diagram

**Fig. 1:**
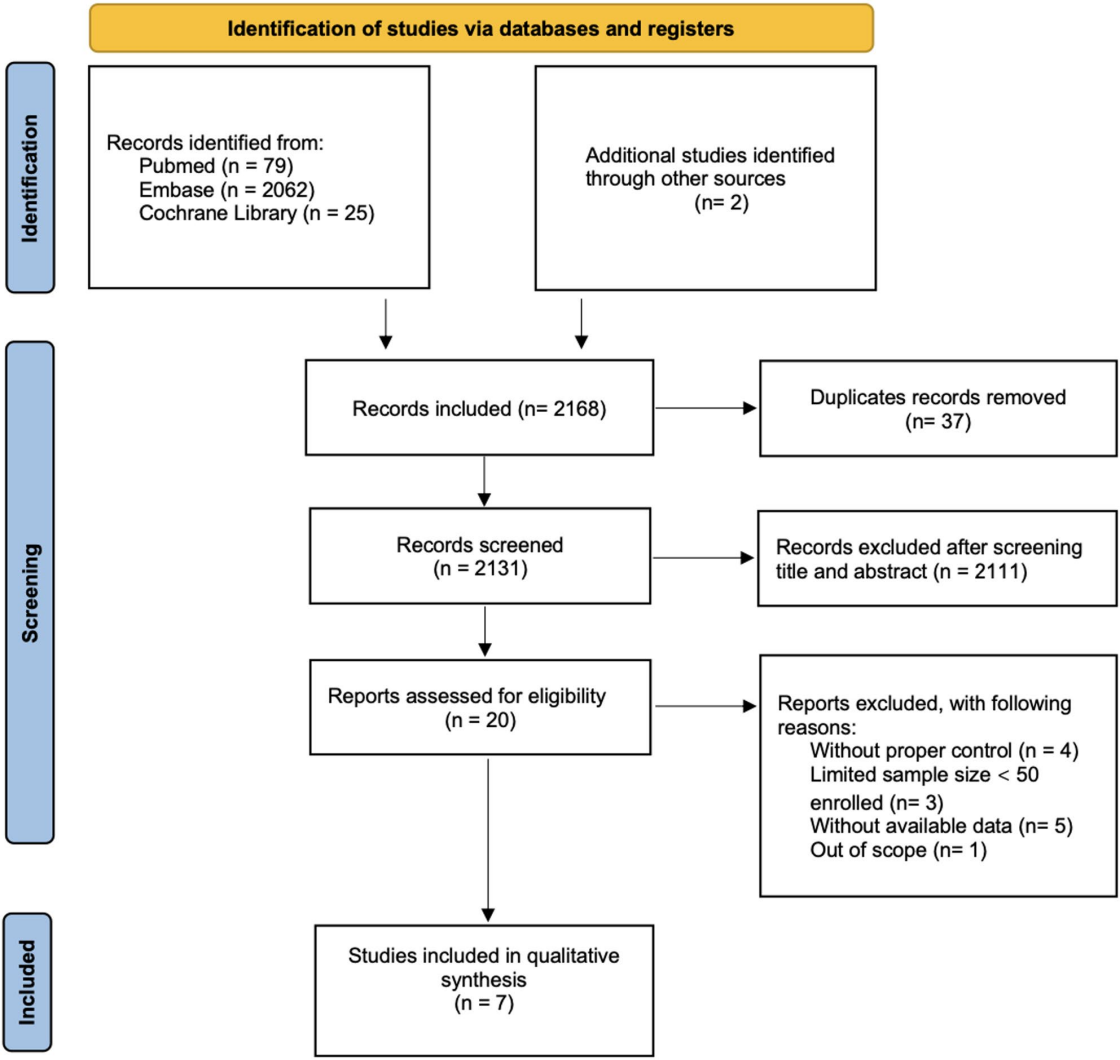
The PRISMA flow diagram

The PRISMA flow diagram is shown in Fig. 1. In total 2166 studies were initially collected from 3 databases (PubMed: 79; Embase: 2062; Cochrane Library: 25) and 2 additional studies from other sources. After the removal of 37 duplicates, 2131 studies were screened by title and abstract, and 2111 were subsequently excluded. Of the 20 studies that underwent full-text assessment, we excluded 13 studies because of without proper control and available data, a limited sample size (< 50), and out of scope. Finally, 7 articles with a total of 6401 participants were included. Of the included studies, 5 were cohort studies, and 2 were cross-sectional studies (Table 1).

**Table 1:** Main characteristics of the included studies assessing the prevalence of triple infection

No. reference	First author (year)	Type of study	Country (Continent)	Sample size, *n*	HBV/HIV/TB triple infection		Male patients, *n* (%)	Age (mean ±SD)	Population enrolled	Quality assessment
					Case, n	Prevalence (%)				
[24]	Kahasit Gebrehiwet (2023)	Cross-sectional study	Ethiopia (Africa)	387	1	0.3	214(55.3)	44.2 ± 15.2	TB	7
[25]	Bonolo B. Phinius (2020)	Cohort study	Botswana (Africa)	300	16	5.3	108(36.0)	NA	HIV	8
[26]	Nokukhanya Msomi (2020)	Cohort study	South Africa (Africa)	4292	130	3.0	1611(37.5)	NA	HIV	7
[27]	Pingzheng Mo (2014)	Cross-sectional study	China (Asian)	361	27	7.5	247(68.4)	39.1 ± 10.1	HIV/TB	7
[28]	R.S.Aires (2012)	Cohort study	Thailand (Asian)	111	3	2.7	289(80.0)	44.1 ± 15.6	TB	6
[29]	Chawin Sirinak (2008)	Cohort study	Argentina (South America)	769	70	9.1	538(70.0)	35.7 ± 8.6	HIV/TB	7
[30]	Maria A. Pando (2007)	Cohort study	Brazil (South America)	181	12	6.6	135(65.9)	34.8 ± 14.1	TB	8

SD: standard deviation; NA = not available

### Prevalence of HIV/TB/HBV triple infection

**Fig. 2:**
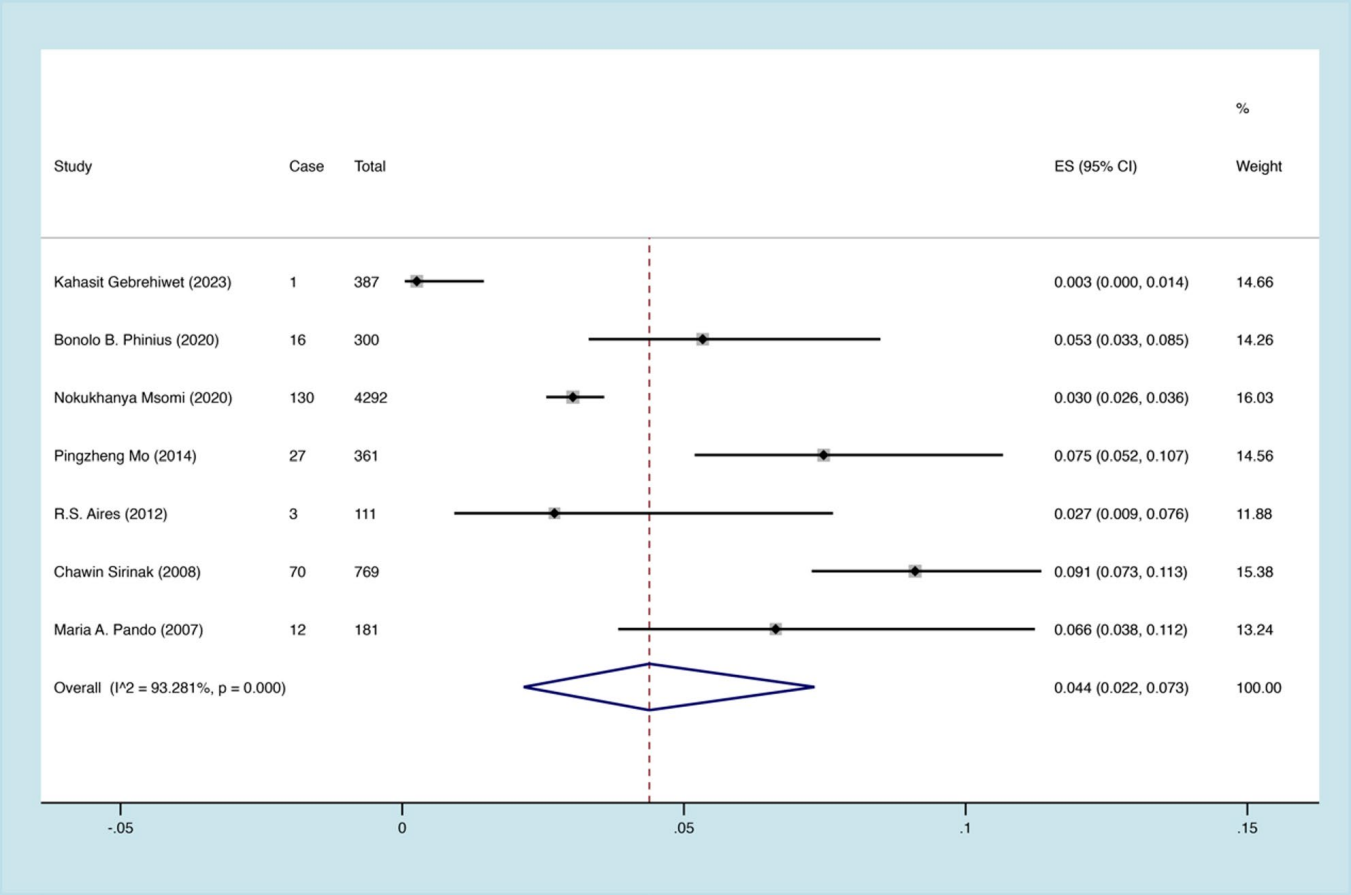
Forest plot showing the overall prevalence of human immunodeficiency virus (HIV), hepatitis B virus (HBV), and tuberculosis (TB) triple infection in the included studies. ES: effect size

**Fig. 3:**
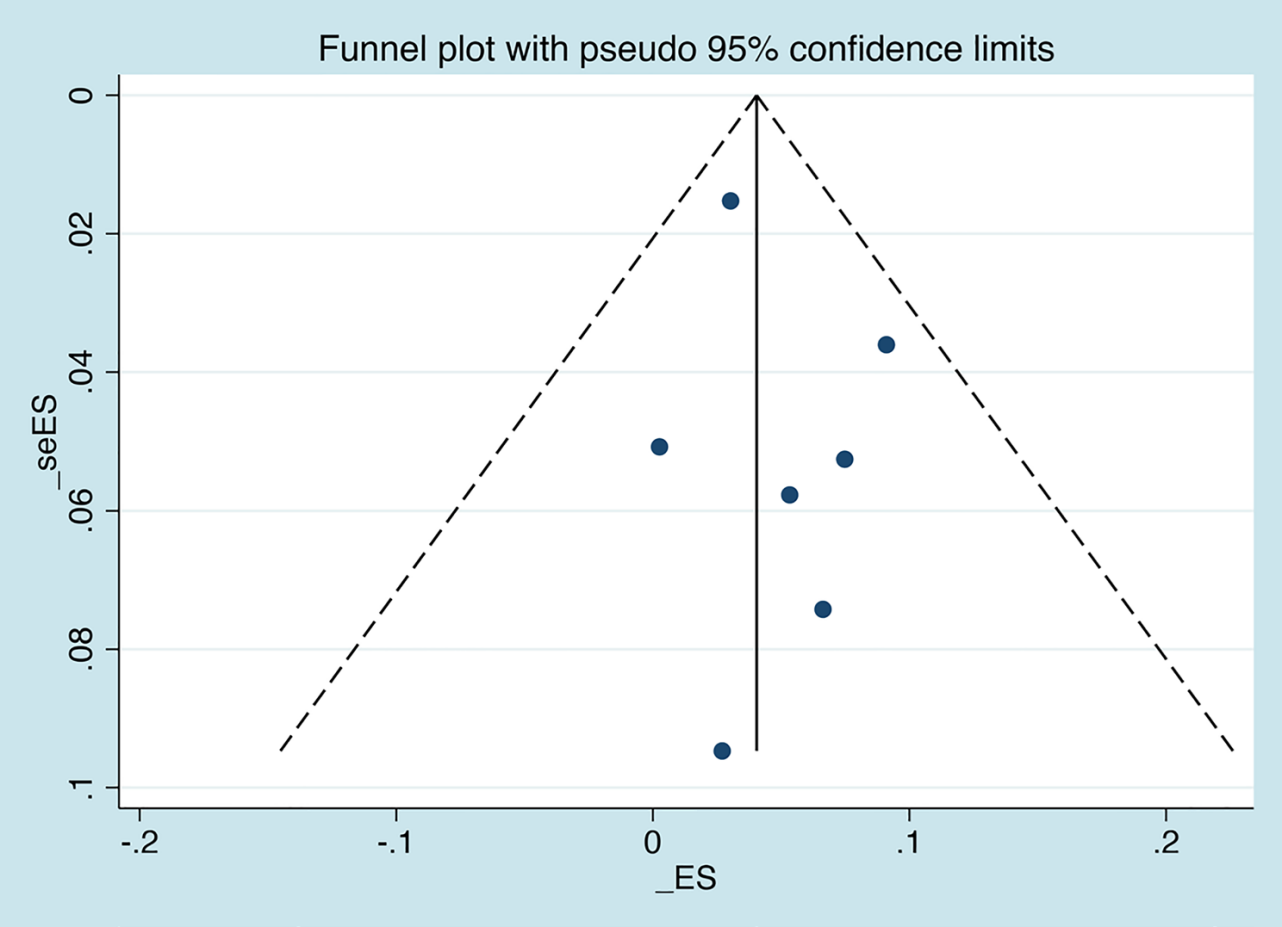
Funnel plot with 95% pseudo confidence limits for all included studies. ES: effect size

**Fig. 4:**
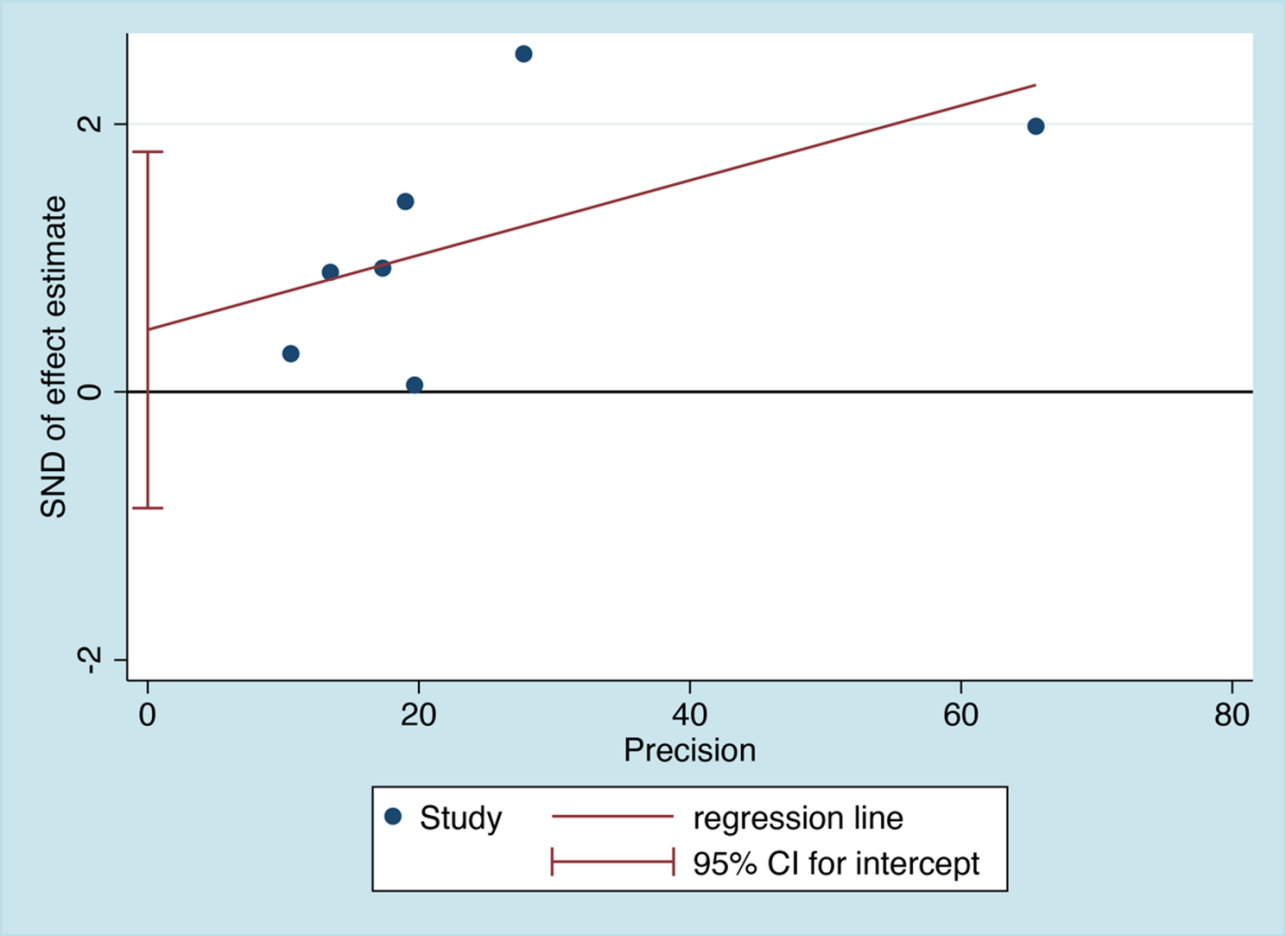
Egger’s test of funnel plot asymmetry with 95% confidence limits and regression line

Within 7 articles included for systematic review and meta-analysis, there were in total 6401 patients enrolled and 259 cases with HIV/TB/HBV triple infection [24–30]. Through pooling the triple infection rates of individual studies included, we initially found that significant heterogeneity was shown among the studies (I^2^ = 98.995%, P <.01) and, therefore, random model was selected for the following calculation process. Finally, it was estimated that the overall prevalence of HIV/TB/HBV triple infection rate was 4.4% (95% CI 2.2%-7.3%) (Fig. 2). In addition, funnel plot and Egger’ test were both employed to exhibit whether or not there were any publication bias in this systematic review and meta-analysis. As demonstrated in Figs. 3 and 4, the funnel plot (Fig. 3) was basically symmetrical, and the Egger test results (Fig. 4) showed no significant statistical evidence of publication bias (t = 0.9, *P* = 0.411).

### Subgroup analysis

In view of the significant heterogeneity of HIV/TB/HBV triple infection rates in different continents, sexes, sample sizes, type of studies, different populations and ART status, subgroup analyses were conducted for these factors (Table 2). Interestingly, we analyzed 7 studies by gender subgroup and found that the triple infection rate was significantly 3-fold higher in man patients (3.4%, 95% CI 0.7%-7.8%) when compared to the female group (1.2%, 95% CI 0.2%-2.9%) (Supplementary Fig. 1). Across Africa, Asian and South America continents, the triple infection rate ranged highest in South America (8.6%, 95% CI 6.8%-10.4%), then Asian next (6.1%, 95% CI 4.1%-8.5%), and Africa last (2.4%, 95% CI 0.5%-5.4%) (Supplementary Fig. 2). Among different countries presented, Argentina exhibited the highest triple infection rate at 9.1% (95% CI 7.3%-11.3%). Due to the lacking reference, triple infection rates of other continents were missing. In addition, population-specific subgroup analyses were performed depending on the characteristics of the populations included in the study, and the results showed that the prevalence of HIV/TB/HBV triple infection was 3.1% (146/4592; 95% CI 2.6%–3.6%) among patients with an initial HIV diagnosis, 2.5% (16/679; 95% CI 0%–8.4%) among those with an initial TB diagnosis, and 8.6% (97/1130; 95% CI 7.0%–10.3%) among patients initially diagnosed with HIV/TB co-infection (Supplementary Fig. 3). Subgroup analysis was also conducted depending on the sample size and type of studies. In studies with a sample size no more than 300, the triple infection rate was higher at 5.1% (31/592; 95% CI 3.4%-7.2%) (Supplementary Fig. 4). Besides, cohort studies reported higher triple infection rate than cross-section studies, with 5.2% (231/5653; 95% CI 2.5%-8.7%) and 2.6% (28/748; 95% CI 1.6%-3.9%) respectively (Supplementary Fig. 5). Finally, we conducted a subgroup analysis comparing patients receiving ART with ART-naïve patients. The results showed the prevalence of HIV/TB/HBV triple infection was higher among patients receiving ART, at 7.4% (125/1611; 95% CI 5.8%–9.2%), compared with 1.7% (134/4790; 95% CI 0.1%–4.6%) in ART-naïve patients. The heterogeneity in this subgroup decreased to I² = 36.269, with a p-value of 0.195, suggesting moderate heterogeneity (Supplementary Fig. 6).

**Table 2:** Prevalence of human immunodeficiency virus (HIV), hepatitis B virus (HBV), and tuberculosis (TB) triple infection in different subgroups

Subgroup	Studies (*n*)	Triple infection, *n* (cases/total)	Prevalence, % (95% CI)	I^2^ (%)	*P* value	
**Pooled prevalence**	7	259/6401	4.4, (2.2–7.3)	98.995	< 0.01	
**Sex**						
male	4	86/1817	3.4, (0.7–7.8)	94.144	< 0.001	
female	4	28/1817	1.2, (0.2–2.9)	83.396	< 0.001	
**Sample size**						
> 300	4	228/5809	4.1, (1.3–8.5)	96.398	< 0.001	
≤ 300	3	31/592	5.1, (3.4–7.2)	-^a^	-^a^	
**Study type**						
cross-sectional study	2	28/748	2.6, (1.6–3.9)	-^a^	-^a^	
cohort study	5	231/5653	5.2, (2.5–8.7)	92.143	< 0.001	
**Continent**						
Africa	3	147/4979	2.4, (0.5–5.4)	-^a^	-^a^	
Asian	2	30/472	6.1, (4.1–8.5)	-^a^	-^a^	
South America	2	82/950	8.6, (6.8–10.4)	-^a^	-^a^	
**Population**						
TB mono-infected	3	16/679	2.5, (0.0-8.4)	-^a^	-^a^	
HIV mono-infected	2	146/4592	3.1, (2.6–3.6)	-^a^	-^a^	
TB/HIV double infected	2	97/1130	8.6, (7.0-10.3)	-^a^	-^a^	
**Treatment**						
ART	4	125/1611	7.4, (5.8–9.2)	36.269	0.195	
ART-naïve	3	134/4790	1.7, (0.1–4.6)	-^a^	-^a^	

-a :Not available; ART: antiretroviral treatment

## Discussion

This systematic review and meta-analysis provides a comprehensive examination of the pooled prevalence of triple infection involving HIV, TB, and HBV. The findings reveal a notable 4.4% prevalence rate globally, shedding light on the significant burden of this triple infection on public health systems worldwide.

In this study, we conclusively reported that the overall prevalence of triple infection was 4.4% (95% CI 2.2%-7.3%). The uncovered triple infection rate of HIV, TB and HBV was lower than that of HIV, HBV and hepatitis D virus (HDV) (7.4%, 95% CI 0.73%-29.59%) [31]. A lower triple-infection rate may be a positive outcome by determination of WHO to eliminate infection of TB and HBV by 2030. Due to the limited data, the triple infection rate in European and North America area was missing. Even though HIV infection rate remained relatively low in western developed countries, some key populations still lived in high risk in HIV infection owing to unprotected sex behavior, shared injecting equipment and illegal drug intake [32, 33].

Kahasit Gebrehiwet et al. reported a triple infection rate as low as 0.26% in Ethiopia [24]. We believed two reasons accounting for that. First, the study was type of cross-sectional one and thus generated point prevalence of the triple infection. Second, the study only included individuals with presumptive TB previously not on ART and/or anti HBV treatment and further excluded those with previously confirmed pulmonary tuberculosis cases who were on treatment or relapse, which may exclude potential cases of triple infection.

Subgroup analysis indicated that South America ranked the highest triple infection rate (8.6%, 95% CI 6.8%-10.4%). Between 2015 and 2023, most countries in South America experienced dramatic increases of TB infection: Paraguay (55.0%), Venezuela (50.0%), Colombia (48.7%), Ecuador (48.4%), Peru (41.8%) [34]. Besides, the larger proportion of high-risk populations in South America, including men who have sex with men (MSM), sex workers, and people who inject drugs and have limited access to healthcare, may further contribute to the elevated prevalence of triple infection [35]. A recent systematic review and meta-analysis has also implied that a high proportion of inmates in Latin America and the Caribbean are infected with HBV or HIV [36]. Nevertheless, our finding was derived from only two available studies, which substantially limits the generalizability of the result. Therefore, additional research with larger and more representative datasets is needed to better clarify the regional burden.

Our subgroup analysis also showed higher triple infection prevalence in men. Biologically, women’s stronger innate and adaptive immunity, influenced by sex hormones, offers protection against infection [37]. Behaviorally, men engage more in high-risk practices—such as injection drug use, hazardous alcohol consumption, and MSM—raising susceptibility to TB, HIV, and HBV infection. Additionally, women’s more frequent contact with healthcare through reproductive services facilitates earlier diagnosis, routine screening, and preventive education, further reducing their risk [38].

We also observed that the prevalence of triple infection was higher in cohort studies compared with cross-sectional ones. This discrepancy can be partly explained by differences in study design. In cohort studies, patients are followed longitudinally, allowing new infections to accumulate during the observation period. By contrast, cross-sectional studies capture prevalence at a single time point and therefore underestimate infections that occur subsequently [39]. This methodological distinction provides a plausible explanation for the higher rates reported in cohort-based analyses in our study.

The higher prevalence of HIV/TB/HBV triple infection observed in patients receiving ART compared with ART-naïve patients may be explained by several factors. First, selection bias could have influenced the findings, as patients eligible for ART are often those with more advanced disease or longer infection duration, increasing their likelihood of acquiring co-infections. Second, ART extends patient survival and follow-up time, thereby increasing the cumulative risk of detecting triple infection [40]. Third, patients on ART are usually subject to more intensive clinical monitoring and diagnostic investigations, which can lead to higher detection rates compared with ART-naïve individuals.

Recognizing the limitations of using the NOS criteria to assess the quality of the included studies, we first examined the case definitions in each study [41, 42]. We found that case definitions were generally adequate, relying on either record linkage or self-reports, which likely minimized the risk of misclassification bias. Besides, a clearly representative sample and detailed description of control selection criteria were provided. To improve the reliability and comparability of future research, it is essential to ensure representative sampling and establish well-defined control group criteria.

There were still certain limitations in our analysis. First, NOS guidelines were selected to fully assess the quality of the included articles and the finally results may be varied depending on the judgement of the reviewers. Second, high heterogeneity was observed among studies included, referring to the variation in study outcomes between studies. We used a random effects model with subgroup analyses whenever possible to reduce the effect of heterogeneity.

While our analysis primarily focused on overall prevalence, it is important to acknowledge that certain high-risk groups—such as sex workers and MSM—are likely to serve as key indicators of HIV/HBV/TB triple infection. Besides, sex and age group were also identified as significant demographic predictors influencing the prevalence of HIV and TB [43].

In conclusion, this systematic review and meta-analysis demonstrates a pooled prevalence of 4.4%, underscoring the significant public health burden posed by HIV, TB, and HBV triple infection in regions with available data. This finding highlights the urgent need for targeted prevention and integrated management strategies to reduce morbidity and mortality associated with triple infection. However, the lack of data from Europe, North America, and western Asia represents a notable limitation, restricting the generalizability of our results and emphasizing the necessity for future studies in underrepresented regions. Although only a small number of eligible studies were identified despite the literature search spanning from 1990 onward, this review serves as an important foundation for summarizing existing evidence and underscores the urgent need for further epidemiological and clinical studies in this underexplored field. Addressing these gaps will be essential for a more accurate global assessment and for guiding policies aimed at mitigating the impact of triple infection while advancing the Sustainable Development Goals related to infectious diseases and health equity. 

## Supplementary Information

Below is the link to the electronic supplementary material.

Supplementary Material 1

Supplementary Material 2

## Data Availability

Data generated in this study is available from the corresponding author upon request.

## Abbreviations

HBsAg: Hepatitis B virus surface antigen
HBV: Hepatitis B virus
HIV: Human immunodeficiency virus
TB: Tuberculosis
HDV: Hepatitis D virus
AIDS: Acquired immunodeficiency syndrome
NOS: Newcastle-ottawa scale
PRISMA: Preferred reporting items for systematic reviews and meta-analyses
AFB: Acid-fast bacillus
ART: Antiretroviral therapy
MSM: Men having sex with men
