# Supplementary material for: Unveiling the underestimated prevalence of HIV, HBV and TB triple infection in Asia, South America, and Africa: a systematic review and meta-analysis
Source: BMC Infect Dis. 2025 Dec 20;26:140. doi: 10.1186/s12879-025-12390-4 (PMC12836920; doi:10.1186/s12879-025-12390-4)
Supplement: Supplementary file 2 — Supplementary Material 2 [file 12879_2025_12390_MOESM2_ESM.pdf]

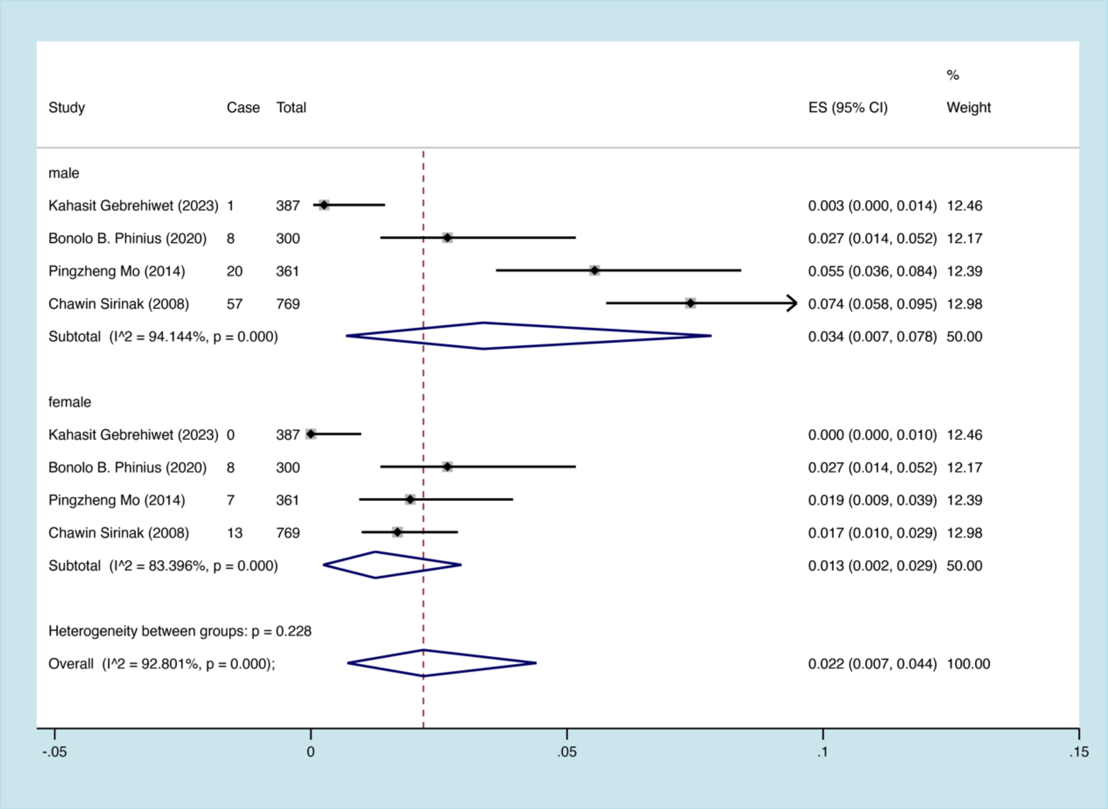

Supplementary Figure 1. Forest plot showing the prevalence of triple infection between different genders.

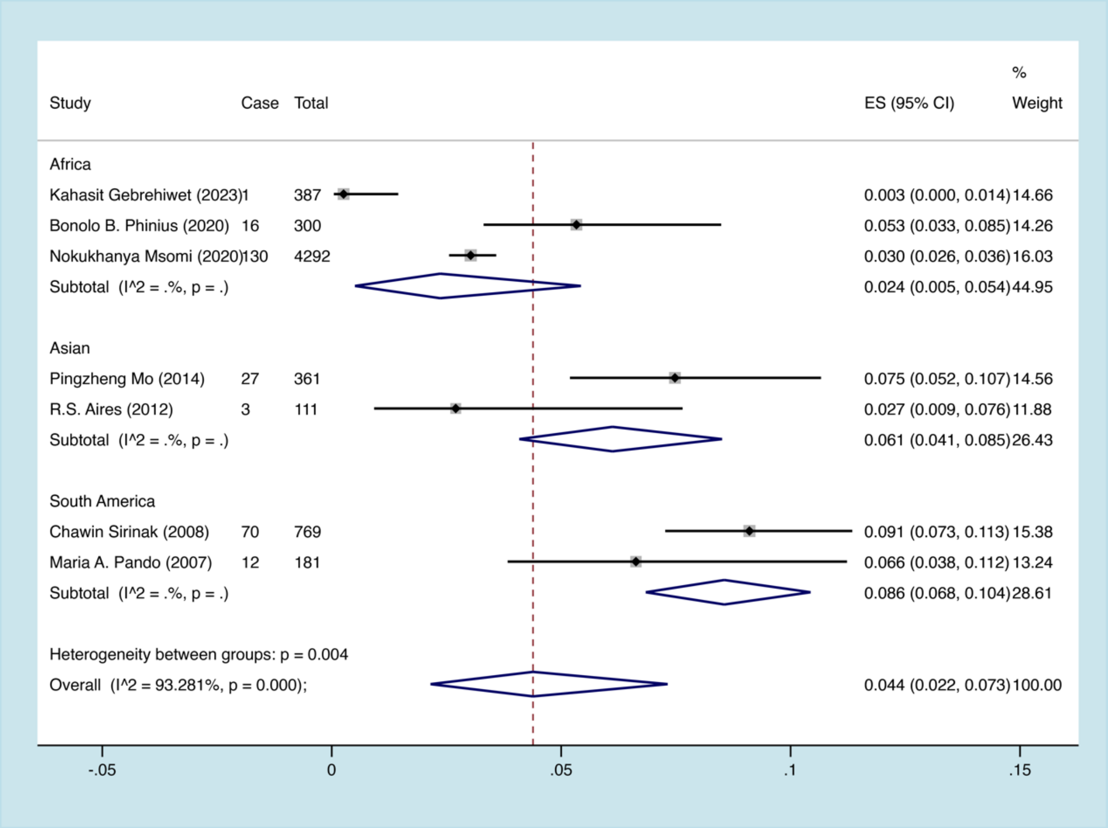

Supplementary Figure 2. Forest plot showing the prevalence of triple infection between different continents.

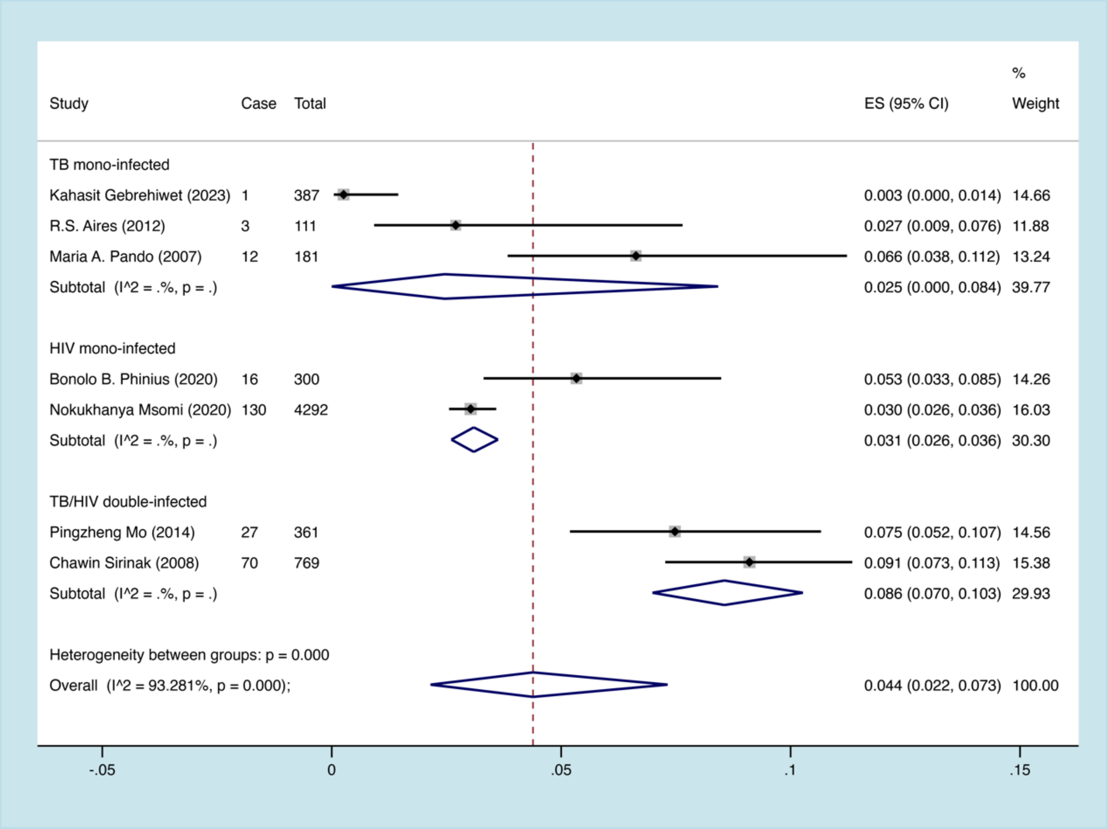

Supplementary Figure 3. Forest plot showing the prevalence of triple infection among different populations.

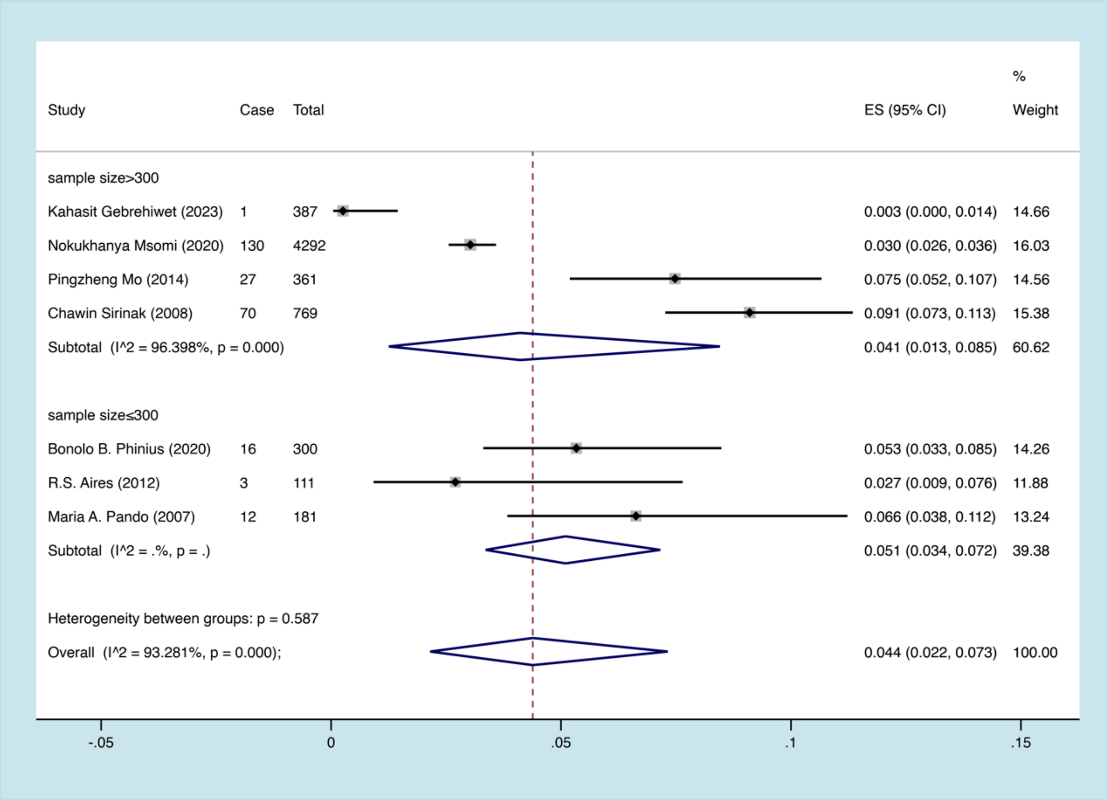

Supplementary Figure 4. Forest plot showing the prevalence of triple infection among different sample sizes.

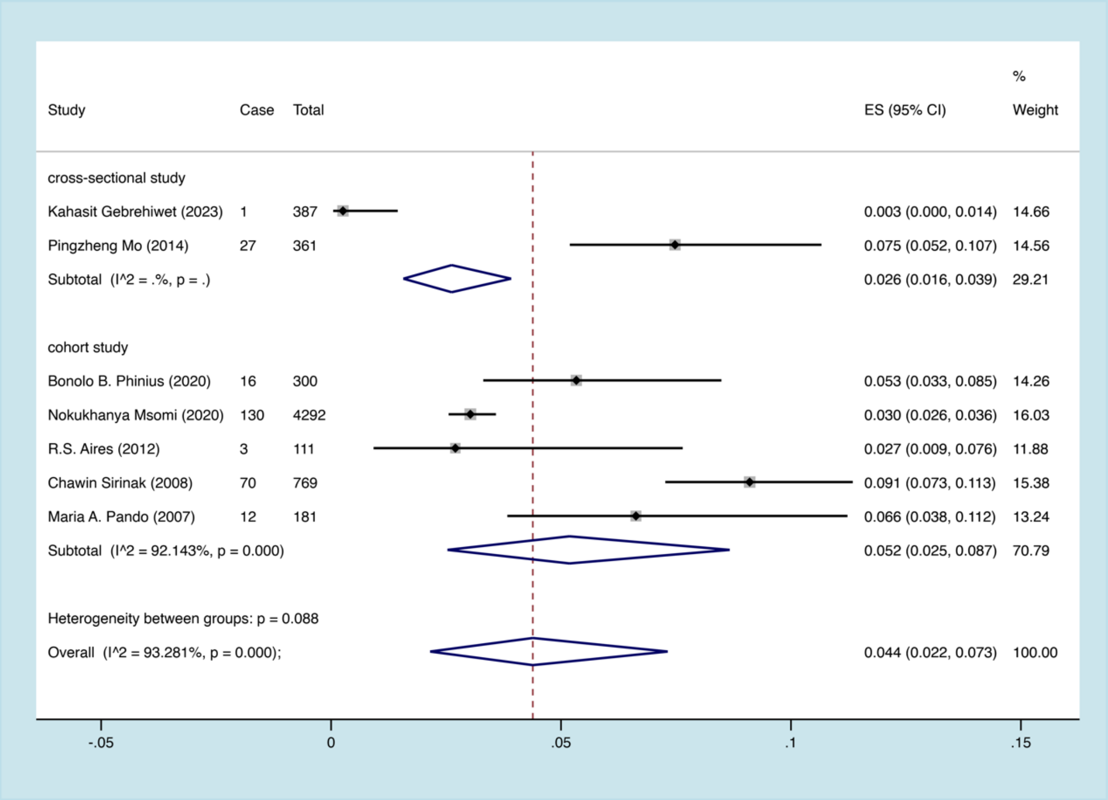

Supplementary Figure 5. Forest plot showing the prevalence of triple infection between different types of study.

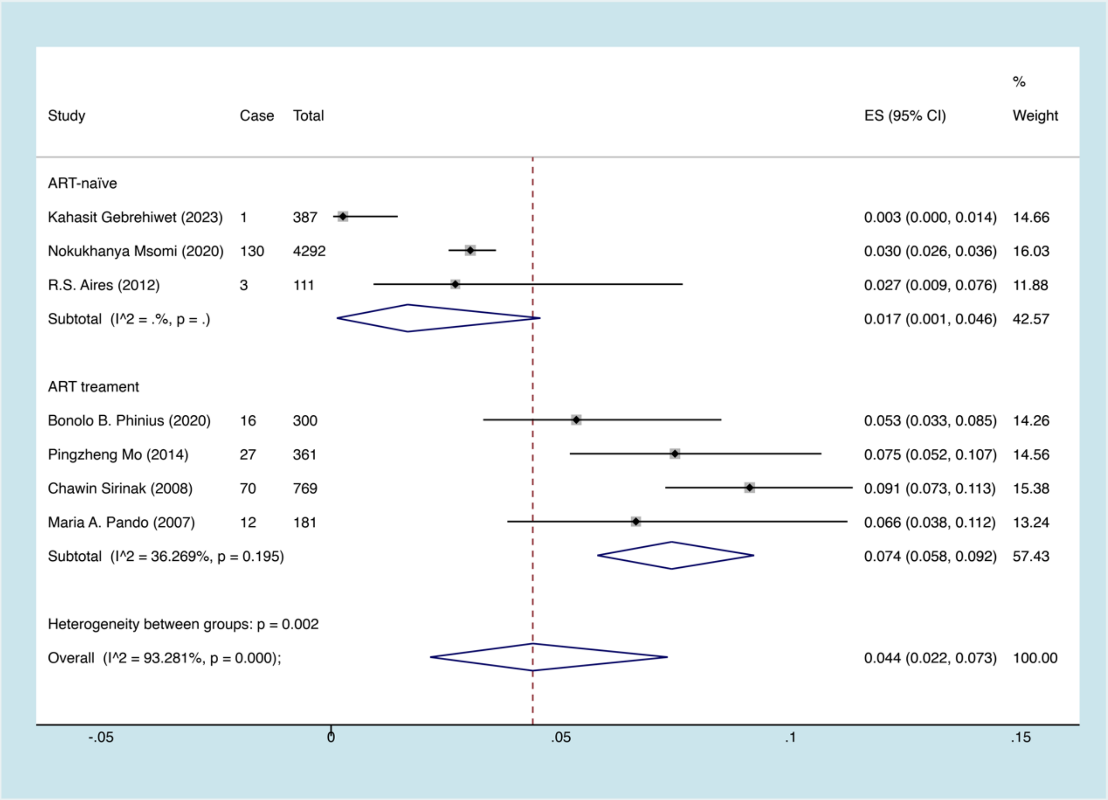

Supplementary Figure 6. Forest plot comparing the prevalence of HIV/TB/HBV triple infection between patients receiving ART and ART-naïve patients. ART: antiretroviral therapy

Supplementary Table 1. Full search strategy applied in this systematic review and meta-analysis.

Database searched	Results (n)	Full search strategy
PubMed	79	((("HIV"[Mesh]) OR (((((((((((((((((((((((((Human Immunodeficiency Virus[Title/Abstract]) OR (Immunodeficiency Virus, Human[Title/Abstract])) OR (Immunodeficiency Viruses, Human[Title/Abstract])) OR (Virus, Human Immunodeficiency[Title/Abstract])) OR (Viruses, Human Immunodeficiency[Title/Abstract])) OR (Human Immunodeficiency Viruses[Title/Abstract])) OR (Human T Cell Lymphotropic Virus Type III[Title/Abstract])) OR (Human T-Cell Lymphotropic Virus Type III[Title/Abstract])) OR (Human T-Cell Leukemia Virus Type III[Title/Abstract])) OR (Human T Cell Leukemia Virus Type III[Title/Abstract])) OR (LAV-HTLV-III[Title/Abstract])) OR (Lymphadenopathy-Associated Virus[Title/Abstract])) OR (Lymphadenopathy Associated Virus[Title/Abstract])) OR (Lymphadenopathy-Associated Viruses[Title/Abstract])) OR (Virus, Lymphadenopathy-Associated[Title/Abstract])) OR (Viruses, Lymphadenopathy-Associated[Title/Abstract])) OR (Human T Lymphotropic Virus Type III[Title/Abstract])) OR (Human T-Lymphotropic Virus Type III[Title/Abstract])) OR (AIDS Virus[Title/Abstract])) OR (AIDS Viruses[Title/Abstract])) OR (Virus, AIDS[Title/Abstract])) OR (Viruses, AIDS[Title/Abstract])) OR (Acquired Immune Deficiency Syndrome Virus[Title/Abstract])) OR (Acquired Immunodeficiency Syndrome Virus[Title/Abstract])) OR (HTLV-III[Title/Abstract]))) AND ((("Hepatitis B"[Mesh]) OR ((Hepatitis B, Chronic[Title/Abstract]) OR (Hepatitis B Virus[Title/Abstract]))) OR (HBV[Title/Abstract]))) AND (("Tuberculosis"[Mesh]) OR (((Tuberculoses[Title/Abstract]) OR (Kochs Disease[Title/Abstract])) OR (Mycobacterium tuberculosis Infection[Title/Abstract])))
Embase	2062	"human immunodeficiency virus'/exp OR 'human immunodeficiency virus' OR 'immunodeficiency virus, human':ab,ti OR 'immunodeficiency viruses, human':ab,ti OR 'virus, human immunodeficiency':ab,ti OR 'viruses, human immunodeficiency':ab,ti OR 'human immunodeficiency viruses':ab,ti OR 'human t cell lymphotropic virus type iii':ab,ti OR 'human t-cell lymphotropic virus type iii':ab,ti OR 'human t-cell leukemia virus type iii':ab,ti OR 'human t cell leukemia virus type iii':ab,ti OR 'lav-htlv-iii':ab,ti OR 'lymphadenopathy-associated virus':ab,ti OR 'lymphadenopathy associated virus':ab,ti OR 'lymphadenopathy-associated viruses':ab,ti OR 'virus, lymphadenopathy-associated':ab,ti OR 'viruses, lymphadenopathy-associated':ab,ti OR 'human t lymphotropic virus type iii':ab,ti OR 'human t-lymphotropic virus type iii':ab,ti OR 'aids virus':ab,ti OR 'aids viruses':ab,ti OR 'virus, aids':ab,ti OR 'viruses, aids':ab,ti OR 'acquired immune deficiency syndrome virus':ab,ti OR 'acquired immunodeficiency syndrome virus':ab,ti OR 'htlv-iii':ab,ti OR 'hiv':ab,ti' AND "hepatitis b'/exp OR 'hepatitis b' OR 'hepatitis b, chronic':ab,ti OR 'hepatitis b virus':ab,ti OR 'hbv':ab,ti' AND "tuberculosis'/exp OR 'kochs disease':ab,ti OR 'mycobacterium tuberculosis infection':ab,ti OR 'tb':ab,ti'

Cochrane Library	25	((Liver Diseases) OR (Disease, Liver OR Liver Dysfunction) OR (liver injury OR hepatic disorder)) AND ((COVID-19) OR (SARS-CoV-2 Infection OR SARS-CoV-2 Infections OR Severe Acute Respiratory Syndrome Coronavirus 2 Infection))

Supplementary Table 2. Newcastle Ottawa Scale for cohort study.

**Selection** (select one star in each section)		
1. Representativeness of the intervention cohort_		
	a) truly representative of the average, elderly, community-dwelling resident	★
	b) somewhat representative of the average, elderly, community-dwelling resident	★
	c) selected group of patients, e.g., only certain socio-economic groups/areas	
	d) no description of the derivation of the cohort	
2. Selection of the non-intervention cohort		
	a) drawn from the same community as the intervention cohort	★
	b) drawn from a different source	
	c) no description of the derivation of the non-intervention cohort	
3. Ascertainment of intervention		
	a) secure record (e.g. health care record)	★
	b) structured interview	★
	c) written self-report	
	d) other / no description	
4. Demonstration that outcome of interest was not present at start of study		
	a) yes	★
	b) no	
**Comparability** (select one or two stars, as appropriate)		
1. Comparability of cohorts on the basis of the design or analysis		
	a) study controls for age, sex, marital status	★
	b) study controls for any additional factors (e.g., socio-economic status, education)	★
**Outcome** (select one star in each section)		
1. Assessment of outcome		
	a) independent blind assessment	★
	b) record linkage	★
	c) self-report	
	d) other / no description	
2. Was follow up long enough for outcomes to occur		
	a) yes, if median duration of follow-up >= 6 month	★
	b) no, if median duration of follow-up < 6 months	
3. Adequacy of follow up of cohorts		
	a) complete follow up: all subjects accounted for	★
	b) subjects lost to follow up unlikely to introduce bias: number lost <= 20%, or description of those lost suggesting no different from those followed	★
	c) follow up rate < 80% (select an adequate %) and no description of those lost	
	d) no statement	

Supplementary Table 3. Adapted Newcastle Ottawa Scale for cross-sectional study.

Module	Item	Criteria for judgment
Selection (maximum 4★)	Representativeness of the sample (Score★: 0–1)	★ Community-based or nationally representative sample;<br>★ High-risk population (e.g., men having sex with man, sex workers, drug users, inmates);<br>☒ Selected group of users;<br>☒ No description of the sampling strategy
	Sample size (Score★: 0–1)	★ Adequate sample size with justification or power calculation;<br>☒ Unjustified
	Non-respondents (Score★: 0–1)	★ Response rate ≥80% or detailed description of non-respondents;<br>☒ Response rate <80% or unsatisfactory comparability between respondents and non-respondents;<br>☒ No description
	Ascertainment of exposure/outcome (Score★: 0–1)	★ Laboratory-confirmed diagnosis (ELISA, PCR, GeneXpert, etc.);<br>★ Available or described medical records;<br>★ Self-reported only<br>☒ No description
Comparability (maximum 2★)	Control for confounders (Score★: 0–2)	★ Study controls for at least one key confounder (e.g., age, sex, treatment, risk group);<br>★★ Study controls for ≥2 confounders
Outcome (maximum 3★)	Assessment of outcome (Score★: 0–1)	★ Independent blind assessment;<br>★ Record linkage;<br>★ Self-report;<br>☒ No description
	Statistical test (Score★: 0–1)	★ Appropriate statistical analysis with confidence intervals, variance, or adjusted prevalence;<br>☒ Inappropriate/incomplete or not described
	Reporting quality (Score★: 0–1)	★ Clear numerator/denominator, prevalence estimates, and study period reported;<br>☒ Missing key information

Supplementary Table 4. STATA commands used in this systematic review and meta-analysis.

Analysis Step	Command
Single proportion meta-analysis	metaprop Case Total, random ftt cimethod(excat) lcols(Study Case Total) dp(3) texts(180)
Subgroup analysis	metaprop Case Total, random ftt cimethod(excat) lcols(Study Case Total) dp(3) texts(160) by(group)
Funnel plot	metafunnel _ES _seES, xtitle(_ES) ytitle(_seES)
Egger’s test	metabias _ES _seES, egger graph
Figure export	graph export ".pdf", replace
